# Preparation of Imazethapyr Surface Molecularly Imprinted Polymers for Its Selective Recognition of Imazethapyr in Soil Samples

**DOI:** 10.1155/2018/7535417

**Published:** 2018-09-30

**Authors:** Yanqiang Zhou, Yinhui Yang, Meihua Ma, Zhian Sun, Shanshan Wu, Bolin Gong

**Affiliations:** ^1^College of Chemistry and Chemical Engineering, North Minzu University, Yinchuan 750021, China; ^2^College of Chemistry and Chemical Engineering, Beijing Institute of Technology, Beijing 100081, China

## Abstract

A novel strategy based on imazethapyr (IM) molecular-imprinting polymers (MIPs) grafted onto the surface of chloromethylation polystyrene resin via surface-initiated atom transfer radical polymerization (SI-ATRP) for specific recognition and sensitive determination of trace imazethapyr in soil samples was developed. The SI-ATRP was performed by using methanol-water (4 : 1, v/v) as the solvent, acrylamide as the functional monomer, trimethylolpropane trimethacrylate (TRIM) as the cross-linker, imazethapyr as the template, and CuBr/2,2′-bipyridine as the catalyst. The resulting MIPs were characterized by elemental analysis, Fourier-transform infrared spectroscopy (FT-IR), scanning electron microscopy (SEM), and transmission electron microscopy (TEM). Then, the binding selectivity, adsorption capacity, and reusability of the MIPs were evaluated. The results indicated that the prepared MIPs exhibited specific recognition and high selectivity for imazethapyr. The MIPs were further used as solid-phase extraction (SPE) materials coupled with high-performance liquid chromatography (HPLC) for selective extraction and detection of trace imazethapyr from soil samples. The results showed that good linearity was observed in the range of 0.10–5.00 *μ*g/mL, with a correlation coefficient of 0.9995. The limit of detection (LOD) of this method was 15 ng/g, and the extraction recoveries of imazethapyr from real samples were in the range of 91.1–97.5%, which proved applicable for analysis of trace imazethapyr in soils. This work proposed a sensitive, rapid, and convenient approach for determination of trace imazethapyr in soil samples.

## 1. Introduction

Imazethapyr, a type of imidazolinones herbicide, belongs to the imidazolinones family of herbicides that are being extensively used in a wide range of cropping systems to enhance crop yields and protect crops from damage by weeds and annual grasses in soybean and peanut [1, 2]. The improper or incorrect use of imazethapyr may lead to the residue and pollution of the soil and groundwater near field crops and also may affect nontargeted plants [3, 4]. In recent years, the deeper toxic mechanisms of imazethapyr revealed that the accumulated imazethapyrs were particularly unfriendly toward animals and humans, which may result in acute or chronic toxicity. In addition, it would affect the transcription of photosynthesis-related genes and inhibit the antioxidant system of the plants [5] and affect the chlorophyll synthesis [6].

To avoid its harmful effects on target and nontarget organisms and to better understand the behavior of imazethapyr in the environment, it is essential to propose a sensitive, rapid and, convenient approach for adsorption of the imazethapyr. The development of analytical methods suitable to fulfill the requirement of determining imazethapyr in soil has attracted widespread interest. The methods for determination of herbicide residues in soil include solid-phase extraction [7], dispersive solid-phase extraction [8, 9], magnetic solid-phase extraction (MSPE) [10], multiple monolithic fiber solid-phase microextraction (MMF-SPME) [11], high-performance liquid chromatography (HPLC) [12], high-performance liquid chromatography with mass spectroscopy (HPLC-MS/MS) [13], gas chromatography coupled with an electron capture detector (GC-ECD) [14], and molecular imprinting [15, 16]. Compared with other methods, molecular imprinting has emerged as a powerful technique [17] for the preparation of tailor-made recognition material owing to its high affinity and specificity [18–21]. Molecularly imprinted polymers (MIPs) have been widely applied to sample preparation for the selective separation and enrichment of trace analytes [22–26]. Traditionally, the MIPs were synthesized through precipitation polymerization, bulk polymerization, and emulsion polymerization [27, 28]. In most cases, traditional MIPs were prepared in organic solvents. However, low imprinted efficiency, slow mass transfer rate, and high hydrophilia of some pesticide residues were restricting the application of MIPs. It is clear that the development and application of selective sorbent phases are still in demand, particularly if they are applied in aqueous conditions. Thus, MIP materials with high sensitivity, selectivity, and versatility are still in demand [29]. Furthermore, the recognition sites of MIPs synthesized by traditional methods are located in the interior of the polymers, which have poor dispersion and serious embedding. As a result, the template molecule has a certain residue when eluting. The MIP product can be used after being crushed and sieved, and the shape of the product is not uniform. The subsequent processing work requires a large amount of time [30, 31]. The surface-initiated atom transfers radical polymerization (SI-ATRP) has been proposed as a new class of controlled living radical polymerization, which possessed a wide range of monomer choice, high selectivity, effectively reduced the embedding of recognition sites, improved adsorption capacity and adsorption efficiency, high mass transfer rate, and high imprinting efficiency [32, 33]. The conditions required for the polymerization reaction were simple and easy to operate, and there are many types of functional monomers that can be selected, water can be used as a medium for the reaction, and a block polymer can be synthesized. Meanwhile, it could be well controlled by SI-ATRP reagent to avoid adverse reactions, such as radical coupling or disproportionation action. Particularly, the polymer segment, template molecular density, and film thickness could be effectively controlled, which is beneficial to increase the adsorption capacity and adsorption efficiency of MIPs [34].

In this study, the imazethapyr surface MIPs on the surface of chloromethylation polystyrene resin were successfully prepared in the binary mixture solvents of methanol-water (4 : 1, v/v) via SI-ATRP. The MIPs were characterized by elemental analysis, Fourier-transform infrared spectroscopy (FT-IR), scanning electron microscopy (SEM), and transmission electron microscopy (TEM). Then, MIP polymers were used as SPE adsorbents coupled with high-performance liquid chromatography (HPLC) to detect a low concentration of imazethapyr in soil samples.

## 2. Materials and Methods

### 2.1. Reagent and Instrument

Polystyrene resin was purchased from Hebei Cangzhou Baoen Chemical Co., Ltd. (Hebei, China). Copper(I) bromide (CuBr), 2, 2′-bipyridyl (BPy), imazethapyr (IM), imazapyr acid, carbendazim, trimethylolpropane trimethacrylate (TRIM, technical grade), and acrylamide (AM) were purchased from Aladdin Reagent (Shanghai, China). Anhydrous zinc chloride, acetonitrile, sulfuric acid, paraformaldehyde, acetic acid (glacial), acetone, and methanol were purchased from Tianjing Damao Chemical Reagent Factory (Tianjing, China). Soil samples were collected from the field and then mixed, dried, grounded and sifted. All other reagents were of analytical grade, and double distilled water was used throughout the experiment. Before use in HPLC analysis, the solution must be filtered through a 0.45 *μ*m nylon filter.

All chromatographic tests were performed using an LC-20AT chromatographic system (Shimadzu, Japan), including two LC-20AT pumps and an SPD-20A UV–VIS detector. FT-IR was performed on FTIR-8400S (Shimadzu, Japan). SEM was performed on JSM-7500F (JEOL, Japan). TEM was measured on HT7700 (Hitachi Ltd., Japan). An elemental analyzer was purchased from the YiLe Man Element Analysis System Company of German (VarioEL III, German). A constant temperature water bath oscillator was purchased from Shanghai Pudong Physical Optics Instrument Plant (SHZ-C, Shanghai, China). A TG16-WS high-speed centrifuge (Centrifuge Factory, China) was used in this experiment, and a TU-1810-type ultraviolet spectrophotometer was purchased from Beijing general instrument Co., Ltd. (Beijing, China).

### 2.2. Preparation of Chloromethylation Polystyrene Resin (CMCPS)

The chloromethylation polystyrene resin (CMCPS) was prepared by following a previously reported protocol [35]. 15.4 g of polystyrene resin, 9.09 g of paraformaldehyde, 20.4 g of anhydrous zinc chloride, 40 mL of 80% sulfuric acid, and 80 mL of glacial acetic acid were placed in three flasks at 50 to 55°C with magnetic stirring. During the reaction, hydrogen chloride gas was continuously bubbled into the three flasks for 12 h. The obtained resin was first washed with deionized water until the pH of the resin to neutral, and then with acetone for a few times. Lastly, it was dried in the vacuum oven at 60°C.

### 2.3. Preparation of Imazethapyr-Imprinted Polymer via SI-ATRP

The imazethapyr-imprinted polymer was prepared via SI-ATRP. Briefly, the template, functional monomer, and cross-linker that were used in this study were imazethapyr, acrylamide (AM), and TRIM, respectively. IM (1.10 g), AM (1.30 g), and TRIM (7.50 mL) were dissolved in 20 mL methanol-water (4 : 1, v/v). This mixture solution was stirred for 2 h at room temperature, leading to the formation of a complex of imprint molecules and monomers. Then, it was purged with the gas of N_2_ for 30 min and was transferred to a flask containing ATRP initiator CMCPS resin (1.30 g), catalyst-CuBr (0.0258 g), and Bpy (0.0562 g). This reaction system was incubated at room temperature under the protection of nitrogen for 24 h. The final product were collected and washed with ethylenediamine tetraacetic acid disodium salt (EDTA) solution (0.1 mol/L), with deionized water, several times with acetone, and were dried in a vacuum oven at 50°C temperature overnight. Then, the MIPs were extracted by using methanol-acetic acid (9 : 1, v/v) solution in a Soxhlet extractor for 24 h to remove the template thoroughly. After that, the MIPs were washed with ultrapure water until neutral. The nonimprinted polymers (NIPs) were prepared by using the same procedure mentioned above, except the addition of a template molecule.

### 2.4. Batch Rebinding Experiment

The adsorption isotherms were obtained by suspending 0.1 g MIPs in 25 mL methanol solution with different imazethapyr concentrations (1∼10 mmol/L). Meanwhile, the adsorption kinetic curves were obtained by detecting the change over time. Adsorption equilibrium of MIPs and NIPs in IM solution was determined by using an initial IM concentration of 8 mmol/L with different adsorption time periods of 1, 2, 3, 4, 5, 6, 8, and 10 h. The binding amount of IM on MIPs was determined as the difference between the total IM amount and the residual amount in the solution by using the UV spectrophotometer at 218 nm. The same experiment steps were carried out for NIPs.

The adsorption capacity *Q* (mg/g) was calculated according to the following:

(1) $$\begin{array}{c} Q=\frac{\left(C_{0}-C_{\text{e}}\right)V}{m}, \end{array}$$

where *C*_0_ (mg/L) and *C*_e_ (mg/L) are the initial and final concentrations of IM in the solution, respectively; *V* (L) is the total volume of the solution; and *m* (g) is the mass of MIPs.

### 2.5. Selective Binding Experiment

**Figure 1:**
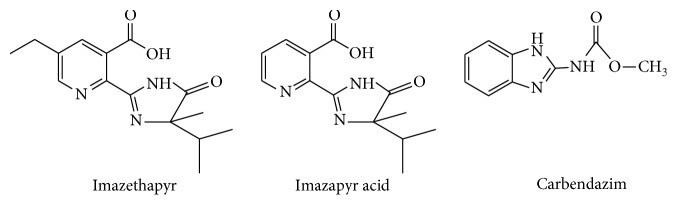


To evaluate the selectivity of MIPs, two structural analogues (schemes), imazapyr and carbendazim, were selected to determine their binding capacities on MIPs and NIPs. 0.1 g of MIPs and NIPs were dispersed in 25 mL of methanol solution containing imazethapyr, imazapyr acid, and carbendazim with an initial concentration of 8 mmol/L. After the adsorption is finished, equilibrium concentrations of each analyte were determined by using a UV spectrophotometer (Figure 1).

The selectivity coefficient of MIPs for imazethapyr with respect to the competition species, imazapyr acid and carbendazim, could be obtained from the equilibrium-binding data according to the following equation:

(2) $$\begin{array}{c} k=\frac{K_{\text{d}1}}{K_{\text{d}2}}, \end{array}$$

where *K*_d1_ represents the distribution coefficient (L/g) of imazethapyr, *K*_d2_ represents the distribution coefficient (L/g) of imazethapyr structural analogues imazapyr and carbendazim, and *k* is the selectivity coefficient. The value of *k* allows an estimation of selectivity of MIPs for imazethapyr.

### 2.6. Desorption Experiment

Certain amounts of MIP particles adsorbing imazethapyr in a saturated state were packed into a piece of glass pipe with an internal diameter of 0.8 cm. The bed volume (BV) of the packed column was 2.0 mL. The eluent of methanol-acetic acid (9 : 1, v/v) was allowed to flow gradually through the column at a rate of two bed volumes per 2 minutes (2 BV/2 min) in countercurrent manner. The effluent with two-volume (2 BV) interval was collected, and the concentration of imazethapyr was determined by UV spectrophotometry at 218 nm. The dynamic desorption curve was plotted, and elution property of MIPs was examined.

### 2.7. Preparation of Imazethapyr MIPs Solid-Phase Extraction Column

An empty polypropylene solid-phase extraction column prepared for the experiment was blocked by a polyethylene sieve plate, and 500 mg of MIP powder was filled into the column, another polyethylene sieve plate was used to compact the MIP powder. The packed columns were washed by methanol-acetic acid (9 : 1, v/v) to remove the template, and the effluent was collected for HPLC detection until IM became undetectable, showing that the template had been cleaned. Then, the residual solvent was removed by methanol and dried for subsequent use. Before each use, 5 mL methanol and deionized water were used to activate the solid phase extraction column. The analytes were eluted by methanol and ammonium hydroxide with a volume ratio of 9 : 1 at each step. The elutions were evaporated until drying and redissolved with 1 mL methanol for further HPLC analysis.

### 2.8. Analysis of Soil Sample

Ten grams of soil sample, 25 mL of 0.1 mol/L ammonium chloride, and ammonia buffer (pH 10) were added to a 50 mL centrifuge tube. The mixture was vortex mixed for 5 min, ultrasonicated for 20 min, and then centrifuged at 6000 r/min for 4 min. The supernatant was collected. This procedure was repeated thrice. The pH of the combined supernatant was adjusted to 2.0 with 1.0 mol/L HCl and volatilized to dry with a rotary evaporator at 45°C. Then, the residue was redissolved into 5 mL methanol, diluted with water to 15 mL, and applied to MIP or NIP cartridges. After SPE, the elution was concentrated to 1 mL and analyzed on the HPLC.

## 3. Results and Discussion

### 3.1. Preparation of MIPs

**Figure 2:**
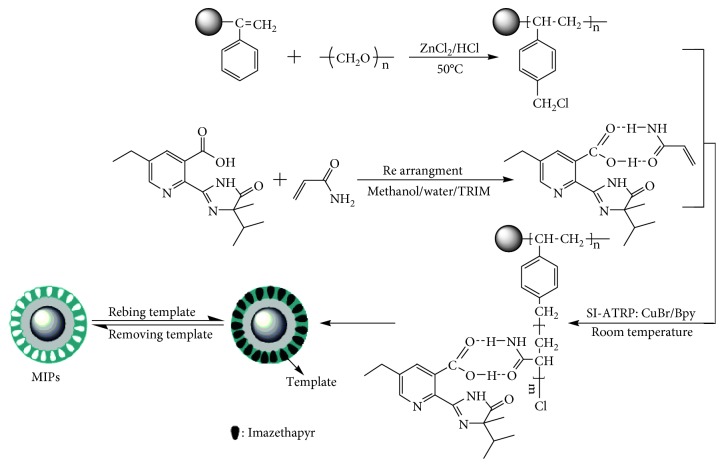
Process for preparing MIPs of imazethapyr.

The preparation procedures for MIPs of imazethapyr were described in Figure 2. The functional monomer initially formed complexes with the template molecules. Then, their functional groups were held in position by the highly cross-linked polymeric structure after polymerization. The MIPs were finally grafted onto the surface of the CMCPS resin via SI-ATRP. After template removal, specific binding sites were left in the polymer material.

### 3.2. Characterization of the MIPs

#### 3.2.1. Elemental Analysis

Nitrogen, carbon, and hydrogen elemental analysis data of CMCPS resin, MIPs, and NIPs are summarized in Table 1. Obviously, *N* content had a significant increase after the IM imprinted onto the surface of CMCPS resin. The amounts of the grafted functional monomer and cross-linker on CMCPS resin were calculated based on the elemental analysis data by using the following:

(3) $$\begin{array}{c} \text{GR}=\frac{{\text{N}}_{\text{i}}\%\times 10^{6}}{28\times \left(1-\text{C}\%-\text{H}\%-\text{N}\%\right)\times S}, \end{array}$$

where C%, H%, and N% are the weight fractions of carbon, hydrogen, and nitrogen of MIPs in grafted imazethapyr on CMCPS resin, respectively. N_i_% represents the weight fraction of nitrogen increase over that of CMCPS resin. *S* is the specific surface area of the chloromethylation polystyrene resin. According to Equation (3), the calculated amount of grafted density was 25.14 *μ*mol/m^2^.

#### 3.2.2. FT-IR Characterization of Polystyrene Resin, CMCPS, and MIPs

**Figure 3:**
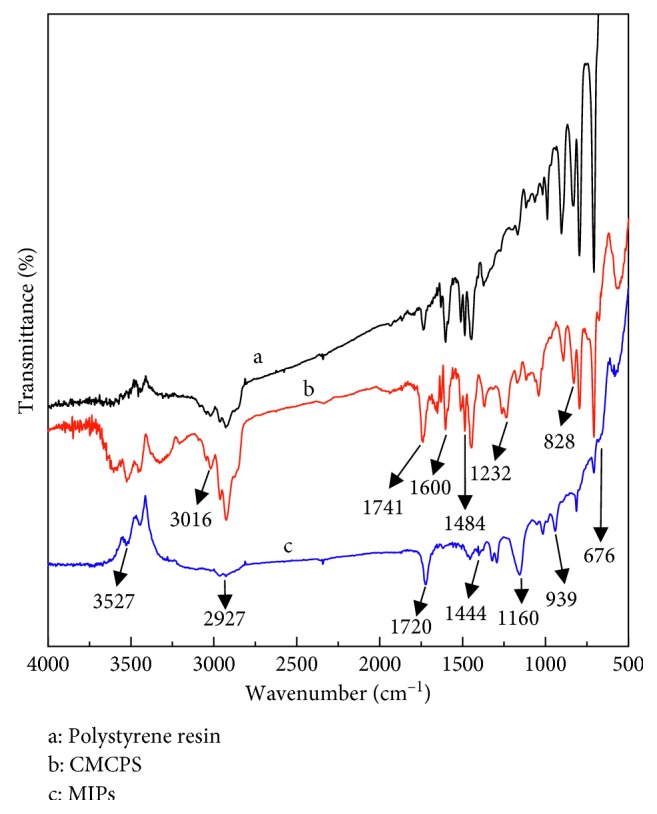
FT-IR spectra of polystyrene resin (a), CMCPS (b), and MIPs (c).

FT-IR spectra of polystyrene resin (a), CMCPS (b), and MIPs (c) are shown in Figure 3. After chloromethylation, the absorption peak changed significantly, of which 676 cm^−1^ is the characteristic absorption peak of -CH_2_Cl, 1232 cm^−1^ is the in-plane bending vibration of -CH bond in -CH_2_Cl, 3016 cm^−1^ is the stretching vibration peak of the -CH bond on the benzene ring, 2958 cm^−1^ and 2927 cm^−1^ are the stretching vibrations of saturated -CH and -CH_2_, respectively. 1741 cm^−1^ and 1720 cm^−1^ are the vibration absorption peaks of -C=O in the aldehyde group. 1484 cm^−1^ and 1444 cm^−1^ are the in-plane and out-of-plane bending vibration peaks of -CH bond on the benzene ring. 828 cm^−1^ is the out-of-plane bending vibration peak of two adjacent hydrogen atoms on the para-disubstituted benzene ring. FT-IR indicated that the H at the 4th position on the cross-linked polystyrene benzene ring has been replaced by -CH_2_Cl, which has successfully produced a chloromethylated polystyrene cross-linked resin.

In MIPs, the increase of peak intensity at 1160 cm^−1^ is due to the stretching vibration of -C-O-C in the cross-linker TRIM. The peak intensity of 3527 cm^−1^ is enhanced because the end of TRIM can react with the exposed hydroxyl groups on the CMCPS, and the number of free hydroxyl groups increases, indicating that the cross-linker TRIM has been successfully polymerized on the surface of the CMCPS.

#### 3.2.3. SEM and TEM Characterization

**Figure 4:**
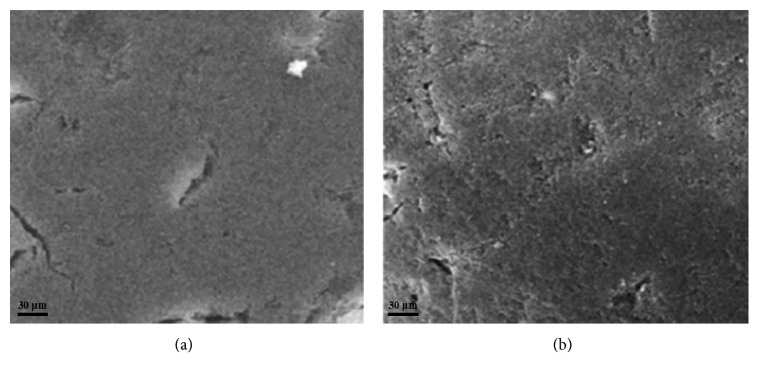
The scanning electron micrographs of (a) CMCPS resin (b) MIPs.

**Figure 5:**
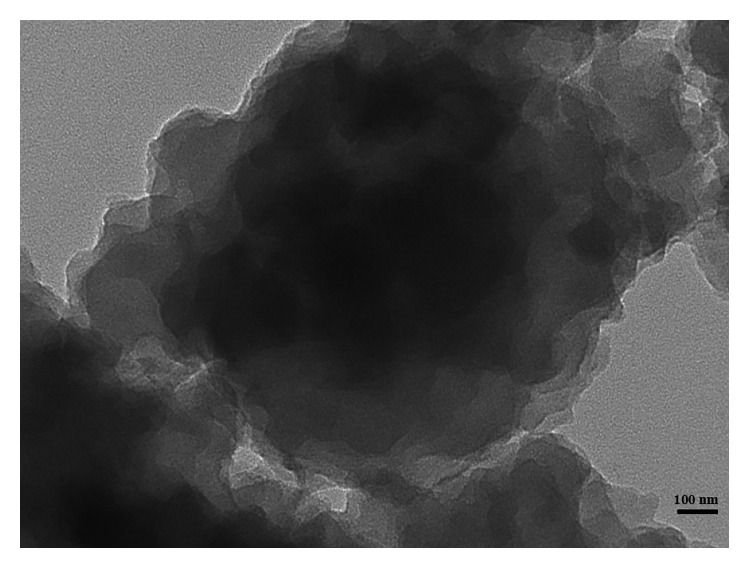
The transmission electron microscope image of MIPs.

SEM and TEM were used to observe the morphology of CMCPS resin and MIPs. As shown in Figures 4(a) and 4(b), the SEM of CMCPS resin exhibited a very smooth and tight surface before imprinting. After imprinting, the MIPs had a rough surface with morphological features and a large number of mesopores, which was beneficial for molecular adsorption and mass transfer. Then, the TEM of MIPs (Figure 5) shows an obvious nuclear-shell structure of MIPs, and particles adhere to each other. The results showed that the imprinted polymer was grafted onto the surface of the CMCPS resin after polymerization and formed a site of interaction with the template molecule.

### 3.3. Effect of the Ratio of Template and Monomer on Adsorption

**Figure 6:**
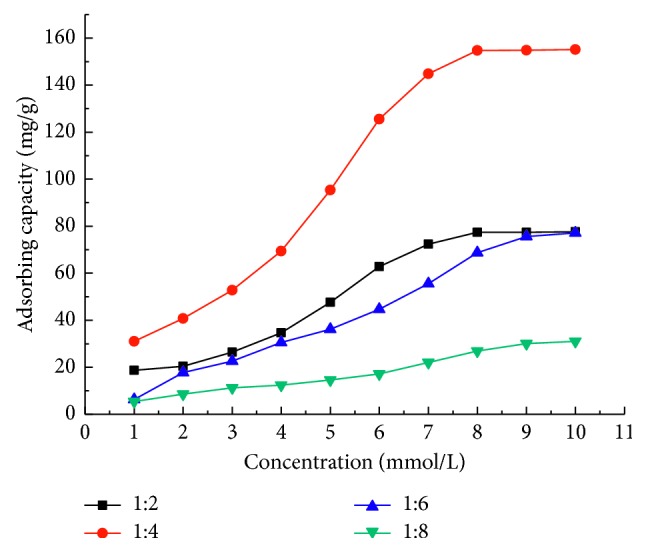
Effect of the ratio of template and monomer on adsorption.

The ratio of template and monomer is one of the most important factors influencing the adsorption behavior of imazethapyr on the MIPs. From Figure 6, the ratios of the template and monomer at 1 : 2, 1 : 4, 1 : 6, and 1 : 8 were selected to investigate the effects. At first, as the concentration of the monomer increased, the adsorption capacity increased significantly. However, when the ratio of the template and monomer was at 1 : 6, the adsorption capacity gradually decreased. It could be that too much of the amount of monomers will enhance nonspecific adsorption capacity, resulting in the reduction of the specific adsorption capacity of MIPs. In this study, the optimal ratio of the template and monomer was at 1 : 4.

### 3.4. Scatchard Analysis

To further estimate the binding property of MIPs, the obtained data were processed with scatchard analysis according to the following equation:

(4) $$\begin{array}{c} \frac{Q}{C_{\text{e}}}=\frac{Q_{\max}}{K_{\text{d}}}-\frac{K_{\text{d}}}{Q}, \end{array}$$

where *Q* and *Q*_max_ are the experimental adsorption capacity to the template imazethapyr (mg/g) and the theoretical maximum adsorption capacity of the polymer (mg/g), respectively; *C*_e_ is the concentration of imazethapyr in equilibrium solution (mg/L); and *K*_d_ is the dissociation constant (mg/L).

**Figure 7:**
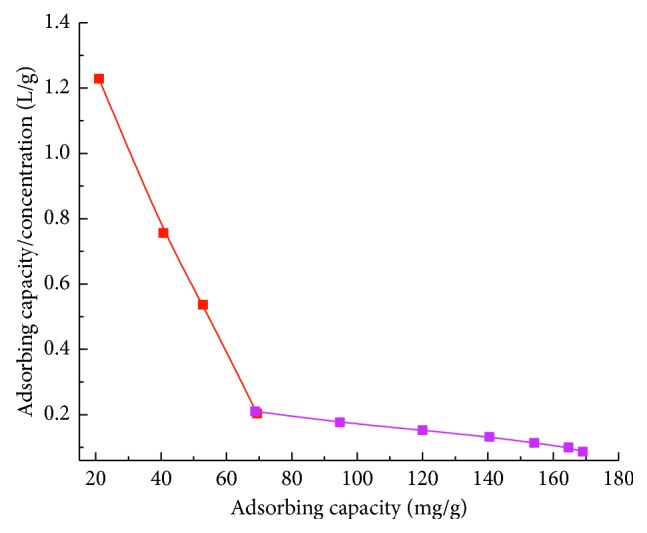
Scatchard's plot for imazethapyr MIPs.

The values of *K*_d_ and *Q*_max_ could be calculated from the slope and intercept of the linear line plotted in *Q/C*_e_ versus *Q*. As shown in Figure 7, the two distinct sections of the Scatchard' plot indicate that the binding sites in the MIPs can be classified into two distinct groups with specific binding properties. The respective *K*_d_ and *Q*_max_ values calculated from the slopes and intercepts of the two linear portions are 47.39 mg/L and 78.21 mg/g for higher affinity sites and 833.3 mg/L and 237.32 mg/g for lower affinity sites, respectively.

The linear relevant fitting equations were *y* = −0.0211*x* + 1.6502 and *R* = 0.9982 for higher affinity sites; *y* = −0.0012*x* + 0.2848 and *R* = 0.9915 for lower affinity sites. These results indicated that MIPs had high specific recognition to the template imazethapyr.

### 3.5. Adsorption Kinetics and Selectivity

**Figure 8:**
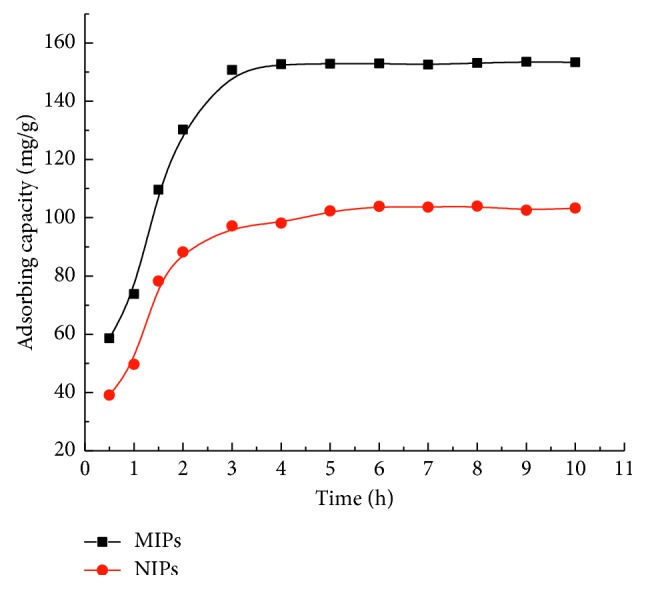
Dynamic adsorptions of MIPs and NIPs.

Figure 8 shows the rapid rebinding rate of MIPs to the template molecule imazethapyr and a sharp increase in the adsorption capacity on MIPs in the first 3 h due to the presence of a large amount of empty and high-affinity binding sites on the surface of the MIPs. After 3 h, the adsorption rate slowed down, and the equilibrium binding of imazethapyr on the MIPs could be reached within approximately 3 h. Clearly, the surface-imprinted MIPs required a much shorter equilibrium time than the traditionally referenced polymerization method. Without the imprinting process, the functional groups were distributed randomly in NIPs, resulting in the low adsorption ability of imazethapyr. The nonspecific adsorption of imazethapyr was observed in NIPs. The results indicated that the prepared MIPs had high specific recognition to the template imazethapyr.

**Figure 9:**
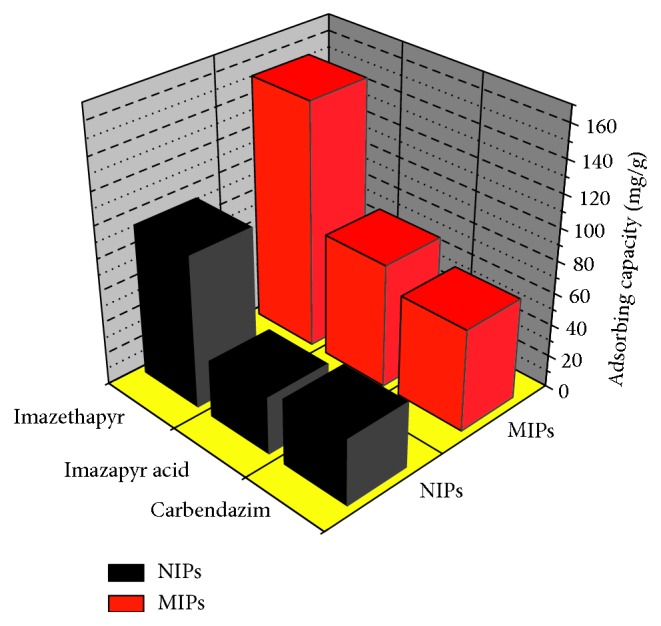
Selective adsorptions for the imazethapyr, imazapyr, and carbendazim.

The adsorption selectivity is an indispensable factor for appreciating capacities of an adsorbent. To evaluate the selectivity of the MIPs, analogues of imazethapyr, imazapyr, and carbendazim were selected. The experimental results were plotted in Figure 9, which showed MIPs a much higher selectivity to imazethapyr than NIPs. Selectivity coefficients for imazapyr imprinted material and nonimprinted material to imazethapyr were 4.26 and 2.12, respectively. They were 3.17 and 1.59 for carbendazim, respectively. Although the structures of imazapyr and carbendazim are very similar with template imazethapyr, the MIPs could still specifically recognize imazethapyr based on the imprinted sites. The results further demonstrate the high selectivity of the MIPs.

### 3.6. Desorption Property

**Figure 10:**
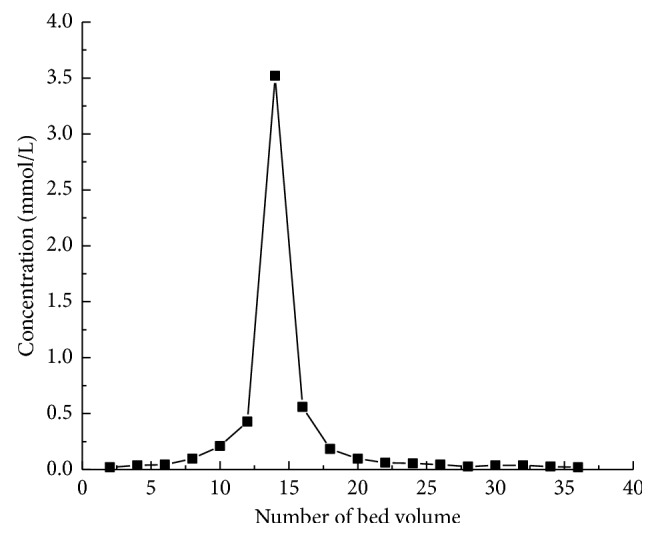
Elution curves of imazethapyr on MIPs.

The dynamic desorption curve of the MIPs adsorbing imazethapyr in a saturated state is shown in Figure 10. This desorption curve was cuspidal without trail formation. The result showed that the desorption ratios in 22 BV and 24 BV reach 93.28% and 97.92%, respectively, which indicated that combined imazethapyr on the MIPs was easy to be desorbed, namely, the MIPs had an excellent eluting property. It was convenient to reuse MIPs.

### 3.7. Validation of the MIPs-SPE Procedure

To evaluate the suitability of the proposed sample preparation technology based on the combination of MIPs and SPE techniques, a method for the analysis of imazethapyr in soil via HPLC was developed. Regression equations, correlation coefficient (*r*), linearity range, limits of detection (LOD), and limit of quantification (LOQ) were investigated.

Matrix-matched calibration standard was applied to eliminate matrix interference effects for the more accurate results. Good linearity (*R* > 0.9995.) was obtained in the range of 0.10–5.0 *μ*g/mL of imazethapyr. The LOD at a signal-to-noise ratio of 3 and LOQ at a signal-to-noise ratio of 10 was 15.0 and 50.0 ng/g, respectively. Then, the recovery tests of MIPs were performed at three spiked levels (0.050, 0.500, and 1.00 *μ*g/g) to evaluate the accuracy of the method. As shown in Table 2, the recoveries of imazethapyr in the spiked samples were in the range of 91.1–97.5%, with relative standard deviation (RSD) in the range of 3.7%–6.5%, indicating that the MIPs can be used for the selective enrichment of imazethapyr in soil. The results fulfilled the demands for analysis. Then, recoveries showed no significant decrease after the MIPs-SPE was used for more than 10 times, indicating its good stability, reusability, and potential for practical analysis.

**Figure 11:**
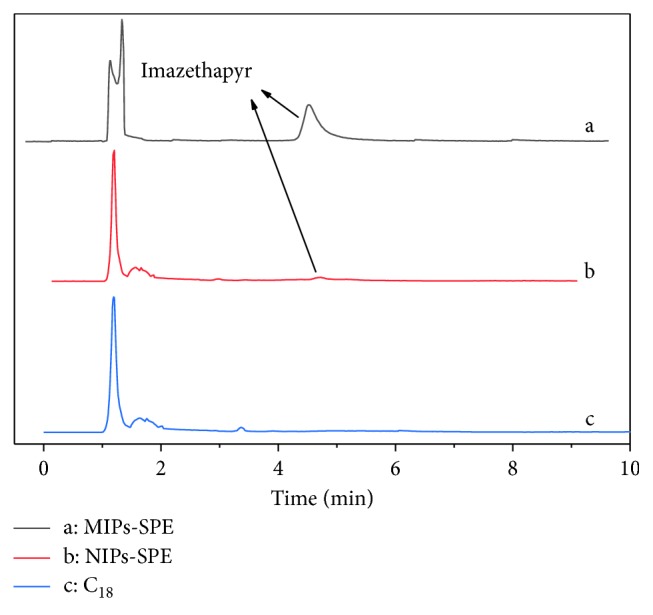
The chromatograms of soil samples with spiked imazethapyr (0.05 *μ*g/g) for MIPs-SPE cartridge (a), NIPs-SPE cartridge (b), and C_18_-SPE cartridge (c).

Taking into account the difficulties of this residue determination and the strong matrix effects that affect this type of analysis, performing a validation in a given soil by different SPE adsorbents demonstrated the method's applicability. To this aim, the use of MIPs, NIPs, and C_18_ is the best way to give realistic information on the method's performance. In this study, the prepared 500 mg MIPs, NIPs, and C_18_ were applied as the SPE adsorbent for the analysis of imazethapyr in the same soil from Yinchuan (Ningxia, China). The chromatograms spiked with 0.050 *μ*g/g of imazethapyr after treating with the MIPs-SPE, NIPs-SPE, and C_18_-SPE columns are shown in Figures 11–11 at the same HPLC test conditions. In terms of the separation of chromatogram peaks, the consequence of using MIPs was better than that of NIPs and C_18_. As shown in Figure 11, the imazethapyr showed a better extraction effect and a higher chromatographic peak than Figure 11, which demonstrated its highly selective recognition of MIPs. However, no target compound was detected but a lot of matrix interference peaks after C_18_ were extracted (Figure 11), which further indicated that MIPs could enhance the enrichment efficiency and selectivity of the extraction and analysis.

### 3.8. Analysis Conditions of HPLC

The flow rate was 1.0 mL/min, and the detection wavelength was set at 258 nm (the maximal absorbance wavelength of imazethapyr). All the separation and detection were performed on the Diamonsil C_18_ column (150 × 4.6 mm). The mobile phase was acetonitrile-0.5% acetic acid glacial (40 : 60, *V/V*).

## 4. Conclusions

In this work, we prepared MIPs using SI-ATRP with imazethapyr as the template and investigated the recognition properties of the MIPs. MIPs possessed excellent recognition ability and combining selectivity for imazethapyr. However, the combining capacity of MIPs for the contrast substances, imazapyr and carbendazim, whose structures were closely similar to imazethapyr, was low. The experimental results revealed that this material could be successfully used as absorbents of SPE coupled with HPLC to separate, enrich, and detect the trace imazethapyr in soil samples. Furthermore, recoveries showed no significant decrease after the MIPs-SPE was used for more than 10 times, which showed that MIPs had good stability and reusability. This study demonstrated that the MIPs were ideal materials in concentration and purification of trace imazethapyrin complex materials and also provides a model for qualitative and quantitative analysis of imazethapyrin in other complex matrices samples. In addition, the method in this study has provided important reference for further monitoring studies to improve soil safety.

## Figures and Tables

**Table 1:** Element analysis of the MIPs.

Samples	Elemental composition (%)		
	N	C	H
CMCPS resin	0.158	80.5	6.54
MIPs	0.289	80.8	6.65
NIPs	0.173	80.7	6.73

**Table 2:** Recoveries of imazethapyr in soil samples.

Sample	Spiked conc. (*μ*g/g)	Measured conc. (*μ*g/g)	Repeatability (RSD%, *n*=3)	Recovery (%)
Soil	0.050	0.0455	6.5	91.1
	0.500	0.487	3.7	97.5
	1.00	0.955	5.8	95.5

## Data Availability

The data used to support the findings of this study are available from the corresponding author upon request.
